# Development of theranostic dual-layered Au-liposome for effective tumor targeting and photothermal therapy

**DOI:** 10.1186/s12951-021-01010-3

**Published:** 2021-09-04

**Authors:** Miyeon Jeon, Gaeun Kim, Wooseung Lee, Seungki Baek, Han Na Jung, Hyung-Jun Im

**Affiliations:** 1 Department of Applied Bioengineering, Graduate School of Convergence Science and Technology, Seoul National University, Seoul, 08826 Republic of Korea; 2 Department of Molecular Medicine and Biopharmaceutical Sciences, Graduate School of Convergence Science and Technology, Seoul National University, Seoul, 08826 Republic of Korea; 3 Cancer Research Institute, Seoul National University, 03080 Seoul, Republic of Korea

**Keywords:** Photothermal therapy, Au-liposome, Hyperthermia, Theranostic, Double-layered liposome

## Abstract

**Background:**

Photothermal therapy (PTT) is an emerging anti-cancer therapeutic strategy that generates hyperthermia to ablate cancer cells under laser irradiation. Gold (Au) coated liposome (AL) was reported as an effective PTT agent with good biocompatibility and excretory property. However, exposed Au components on liposomes can cause instability in vivo and difficulty in further functionalization.

**Results:**

Herein, we developed a theranostic dual-layered nanomaterial by adding liposomal layer to AL (LAL), followed by attaching polyethylene glycol (PEG) and radiolabeling. Functionalization with PEG improves the in vivo stability of LAL, and radioisotope labeling enables in vivo imaging of LAL. Functionalized LAL is stable in physiological conditions, and ^64^Cu labeled LAL (^64^Cu-LAL) shows a sufficient blood circulation property and an effective tumor targeting ability of 16.4%ID g^−1^ from in vivo positron emission tomography (PET) imaging. Also, intravenously injected LAL shows higher tumor targeting, temperature elevation in vivo, and better PTT effect in orthotopic breast cancer mouse model compared to AL. The tumor growth inhibition rate of LAL was 3.9-fold higher than AL.

**Conclusion:**

Based on these high stability, in vivo imaging ability, and tumor targeting efficiency, LAL could be a promising theranostic PTT agent.

**Graphic Abstract:**

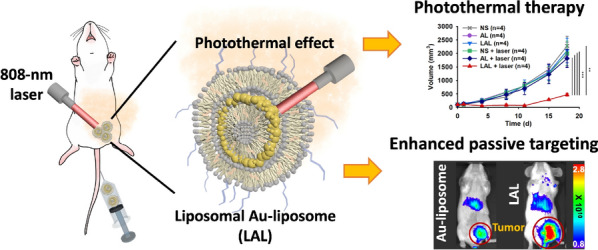

**Supplementary Information:**

The online version contains supplementary material available at 10.1186/s12951-021-01010-3.

## Background

Photothermal therapy (PTT) is one of the rising cancer therapeutics which utilizes the combination of photo-absorbers and near-infrared (NIR) light irradiation to generate hyperthermia and eliminate cancer cells [1, 2]. PTT has multiple advantages over conventional cancer therapeutics, such as high tumor selectivity, ease of therapeutic dose adjustment, less invasiveness, and low likelihood of resistance [3–6]. The high selectivity of PTT can be achieved both by the specific tumor targeting of photo-absorbers and the localized light administration. In addition, the tumor tissues have an insufficient blood supply and low heat resistance than normal tissues and therefore are more sensitive to PTT [7]. PTT has been proven to effectively treat various animal models of malignant tumors including glioblastoma, breast cancer, lung cancer, and colorectal cancer [8]. Furthermore, a recent pilot clinical trial demonstrated that the intratumorally injected Au-silica nanoshells-mediated PTT was successful for the ablation of prostate cancer in 94% (15/16) of patients without significant adverse events [9].

Gold nanoparticles (Au NPs) are considered one of the most promising photo-absorbers for PTT because of the excellent photothermal conversion efficiency and tunability of the absorption band [10–12]. Due to the unique surface plasmon resonance phenomenon, Au NPs have an unprecedently fast and efficient photothermal conversion than organic photo-absorbers [13]. Also, the absorption band of Au NPs can be easily adjusted to match the emission band of the light source by modifying the size, shape, and compositions of the NPs [14]. However, there are drawbacks of Au NPs, which are (1) low tumor targeting ability due to short circulation time, (2) potential toxicity due to long-term retention in the body system, and (3) difficulty of non-invasive assessment of biodistribution. Although Au is considered an inert and biocompatible material, the accumulation of Au in the body system can lead to considerable health risks [15]. The toxicity of Au NPs increases with dose and time [16–19]. When Au NPs are administered to the body system, serum proteins actively interact with Au NPs to produce a protein corona surrounding Au NPs [20, 21]. As a result, Au NPs are opsonized and easily phagocytosed up by the reticuloendothelial system (RES) [22–26]. This leads to the fast clearance of the NPs from the circulation and low tumor targeting efficacy [27]. Also, Au NPs taken up by RES are not excreted in a reasonable timeframe and may cause toxicity [28]. Therefore, Au NPs with high extractability and efficient tumor targeting ability are highly desired for successful clinical translation of Au NP mediated PTT. In 2015, Rengan et al. [29] synthesized the Au coated liposome (AL) that can be excreted from the system efficiently. Au coated liposome can be excreted from the system because the Au component of the NPs is decorated over the liposome rather than forming a solid core, enabling the break down of the Au decorated liposome in the cells. The authors found that most of the Au components can be excreted within 14 days after intravenous (iv.) injection of the NPs. Also, the NP showed in vivo PTT effect after intratumoral injection of the NPs. However, in the biodistribution study, the NPs were rapidly taken up by RES (52%ID in liver at day 1) after iv. injection, suggesting the limited ability of the NPs to the target tumor. Also, in vivo PTT after iv. injection of AL was not performed [29].

**Scheme 1:**
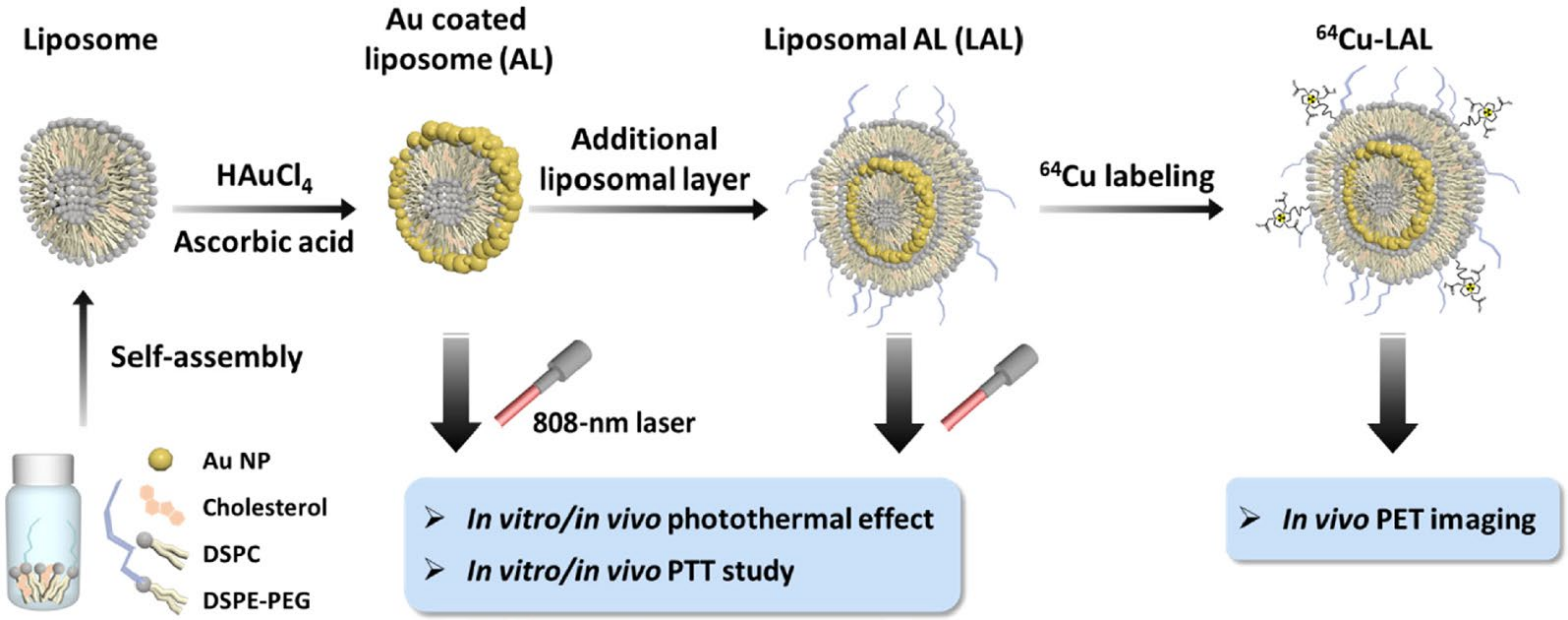
Schematic illustration of experimental procedure of LAL for photothermal therapy (PTT) and ^64^Cu labeled LAL (^64^Cu-LAL) for in vivo imaging

Herein, we developed theranostic dual-layered NPs by adding liposomal layer to AL (liposomal AL, LAL) which has enhanced tumor targeting ability compare to AL (Scheme 1). The NPs are further functionalized by the help of the additional liposome layer; (1) radiolabeling for in vivo imaging enabling theranostics and (2) adding polyethylene (PEG) group to enhance in vivo stability and passive targeting ability. We found that LAL was more stable than AL in vitro and in vivo. LAL has a similarly good photothermal effect with AL. Also, non-invasive quantitative imaging could be done using ^64^Cu-LAL. Intravenously injected LAL showed long circulation time and excellent tumor targeting efficiency by passive targeting (16.4%ID g^−1^) in the orthotopic mouse model of breast cancer. LAL showed 2.9-fold higher in vivo tumor targeting ability than AL based on in vivo fluorescence imaging study. Finally, in vivo tumor growth inhibition rate of LAL mediated PTT was 3.9-fold higher than that of AL mediated PTT (79.4% vs. 20.4%, respectively).

## Results and discussion

### Characterization of LAL and AL

**Fig. 1:**
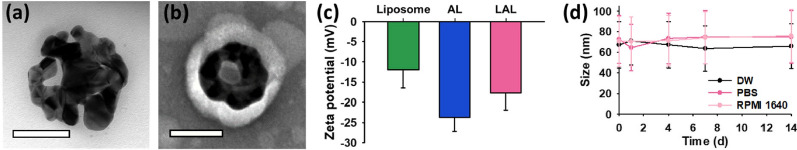
Characterization of AL and LAL. Transmission electron microscopy (TEM) images of **a** AL and **b** LAL. Scale bars are 50 nm. **c** Surface zeta potential values of liposome, AL, and LAL (n = 3, mean ± s.d.). **d** Stability test of LAL in deionized water (DW), phosphate-buffered saline (PBS), and RPMI 1640 with 10% fetal bovine serum (FBS) (n = 3, mean ± s.d.)

The functionalized outer liposomal layer was successfully self-assembled over AL. In transmission electron microscopy (TEM) images, the Au decoration on liposome was observed in the AL sample, and the successfully covered outer liposomal layer over the AL was observed in the LAL sample (Fig. 1a, b). TEM based sizes of AL and LAL were 61.02 ± 29.22 nm and 72.84 ± 22.49 nm, respectively (n = 20, mean ± s.d.). Hydrodynamic sizes of AL and LAL were recorded 3 times in phosphate-buffered saline (PBS) using dynamic light scattering (DLS). Hydrodynamic size of the LAL was 67.32 ± 22.65 nm and the three measurements were almost identical to each other (Additional file 1: Figure S2a). The hydrodynamic size of AL was measured differently in the three repeated measurements: 109.5 ± 52.92 nm, 73.44 ± 41.24 nm, and 49.38 ± 26.95 nm, respectively (Additional file 1: Figure S2b). This could be caused by the instability of AL in PBS. The zeta potential of the inner liposome was − 12.0 mV, which is a typical surface potential of liposomes in the literature [30]. The decreased zeta potential, − 23.7 mV, was observed in AL which can be indirect evidence of successful Au coating to the liposome since bare Au NPs have low zeta potential ranging from − 20 to − 40 mV [23, 31, 32]. As AL covered one more liposomal layer, the zeta potential was elevated to − 17.7 mV because of the PEG moiety of the outer liposomal layer (Fig. 1c). This further confirms successful PEGylation over AL since PEGylation has an impact of lowering negative zeta potential [23, 31–33].

### Size stability of LAL in various solutions

The stabilities of the LAL were tested in various physiological solutions [deionized water (DW), PBS, and cell media with 10% fetal bovine serum (FBS)] to determine the feasibility of in vivo utilization of the LAL. LAL showed no visible aggregates or precipitates and maintained its size ranging from 60 to 80 nm in DW, PBS, and cell media with 10% FBS for 14 days (Fig. 1d, Additional file 1: Figures S1, S2a). On the other hand, AL became unstable in those conditions showing a large range of size variation starting from 24 h after the incubation (Additional file 1: Figure S2b). PEGylated outer liposomal layer increased the stability of LAL diminishing ionic and serum protein interactions. This result suggests that LAL has excellent stability in physiological solutions, which is a prerequisite for in vivo tumor targeting of NPs.

### In vitro photothermal effect of LAL

**Fig. 2:**
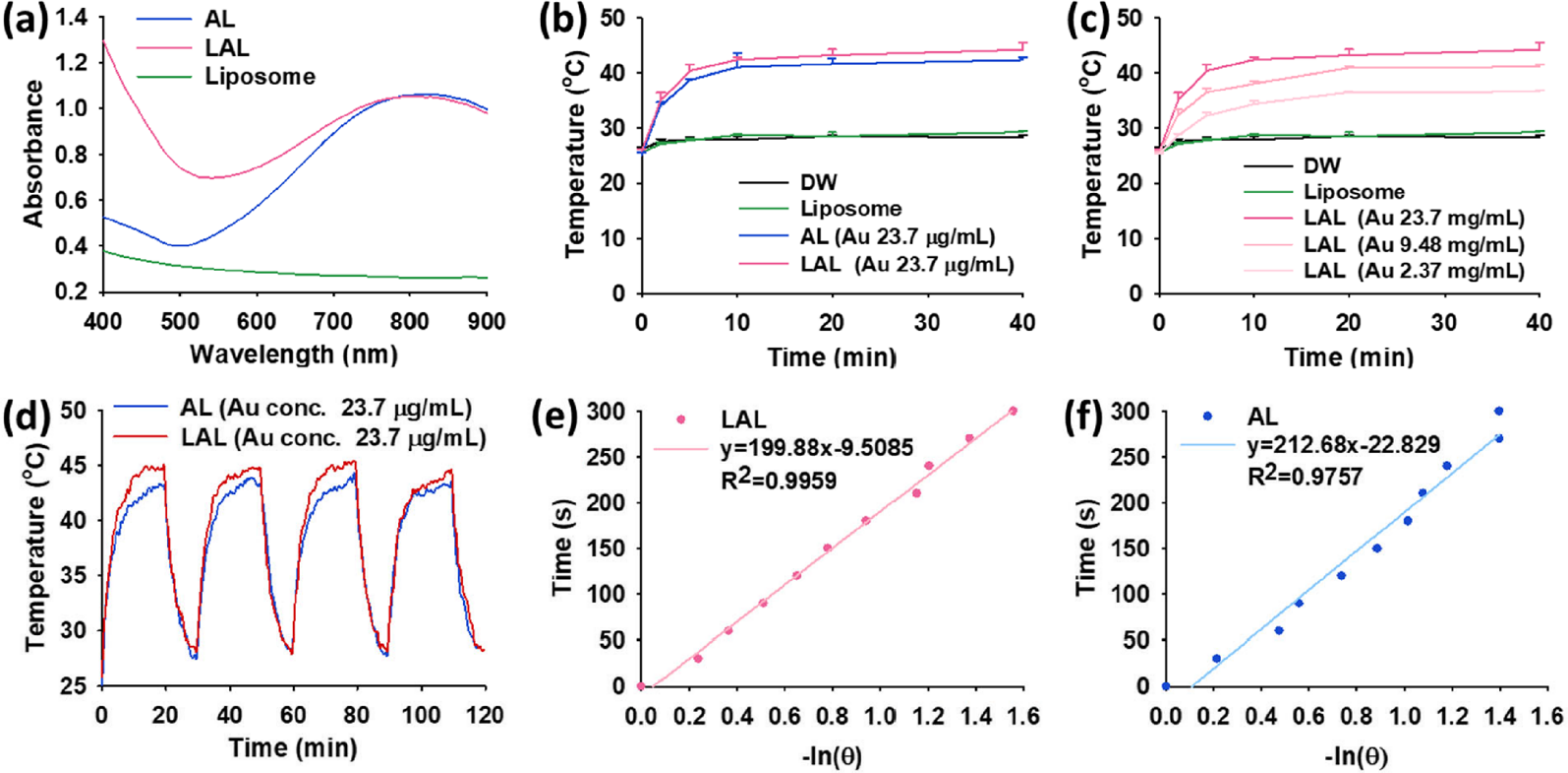
Photothermal effect of AL and LAL. **a** Absorbance spectra of liposome, AL, and LAL. **b** Photothermal effect by observing temperature evaluation of DW, liposome, AL, and LAL under the 808-nm laser irradiation with 1 W intensity for 40 min (n = 3, mean ± s.d.). **c** Au concentration dependent photothermal effect of LAL under the 808-nm laser irradiation with 1 W intensity for 40 min (n = 3, mean ± s.d.). **d** Verification of thermal stability of AL and LAL during the 4 repeats of laser on/off cycle. **e** Time constant for heat transfer of **e** LAL and **f** AL from the photothermal system

Both AL and LAL had a broad absorbance band that starts from 600 nm and has a peak at 900 nm (Fig. 2a). The temperature elevation under the 1 W 808 nm laser irradiation for 40 min presented that AL and LAL had efficient photothermal conversion abilities. The temperatures of the AL and LAL solutions increased from 25.1 ± 0.6 °C to 42.3 ± 0.4 °C, and from 25.8 ± 0.4 °C to 44.2 ± 1.3 °C, respectively, for 40 min (Fig. 2b, Additional file 1: Figure S3). Of note, AL and LAL contained the same Au concentration, 23.7 μg mL^−1^. The temperature of DW and liposome solution without Au slightly increased from 26.2 ± 0.3 °C to 28.2 ± 0.4 °C, and from 25.6 ± 0.6 °C to 29.2 ± 0.3 °C, respectively, under the same laser irradiation condition. After the irradiation, AL and LAL temperature changes were 17.2 ± 0.7 °C and 18.3 ± 1.0 °C, respectively, showing no significance. AL and LAL were similarly effective in photothermal conversion. We also observed that the photothermal effect increased proportionally to the Au concentration in LAL solution (Fig. 2c, Additional file 1: Figure S3). Both AL and LAL showed stable temperature elevation during the four repeats of laser irradiation on/off cycle, allowing them to be used for multiple courses of PTT with single injection in practical applications (Fig. 2d). The photothermal conversion efficiency (*η*) of AL and LAL were calculated by the following Eq. 1 [34, 35].

1 $$\eta =\frac{\mathrm{h}A\left({T}_{\mathrm{max}}-{T}_{\mathrm{sur}}\right)-{Q}_{\mathrm{diss}}}{I(1-{10}^{-A})}=\frac{\mathrm{mc}\left({T}_{\mathrm{max}}-{T}_{\mathrm{sur}}\right)-{Q}_{\mathrm{diss}}}{{\tau }_{s}I(1-{10}^{-A})}$$

where, *T*_max_ is the highest temperature of NP solution, and *T*_sur_ is the initial temperature of NP solution. *Q*_*diss*_ is the heat dissipation, and $I$ represents the power of laser. *A* is the absorbance at 808 nm, *m* is the weight of NP solution, and *c* represents the specific heat capacity of water. *τ*_s_ was determined by Eq. 2.

2 $$\tau_{{\text{s}}} { } = - \frac{t}{ln\theta }$$

where, *θ* refers to the dimensionless driving force, and *t* represents the corresponding time.

Figure 2e, f were used to derive *τ*_s_ values of AL and LAL. The calculated photothermal efficiency values of AL and LAL were 34.13% and 37.46%, respectively, showing that the photothermal efficiency of LAL was slightly higher than AL. These results could be competitive with the photothermal conversion efficiency of other photothermal Au nanomaterials such as Au nanorod (21–22%) [36, 37], Au nanoshell (13%) [38], Au nanofluid (20–21%) [39], Au nanovesicles (18%) [36, 40] and other Au nanomaterials (30–31%) [37, 41].

### Excellent in vitro PTT effect of LAL

**Fig. 3:**
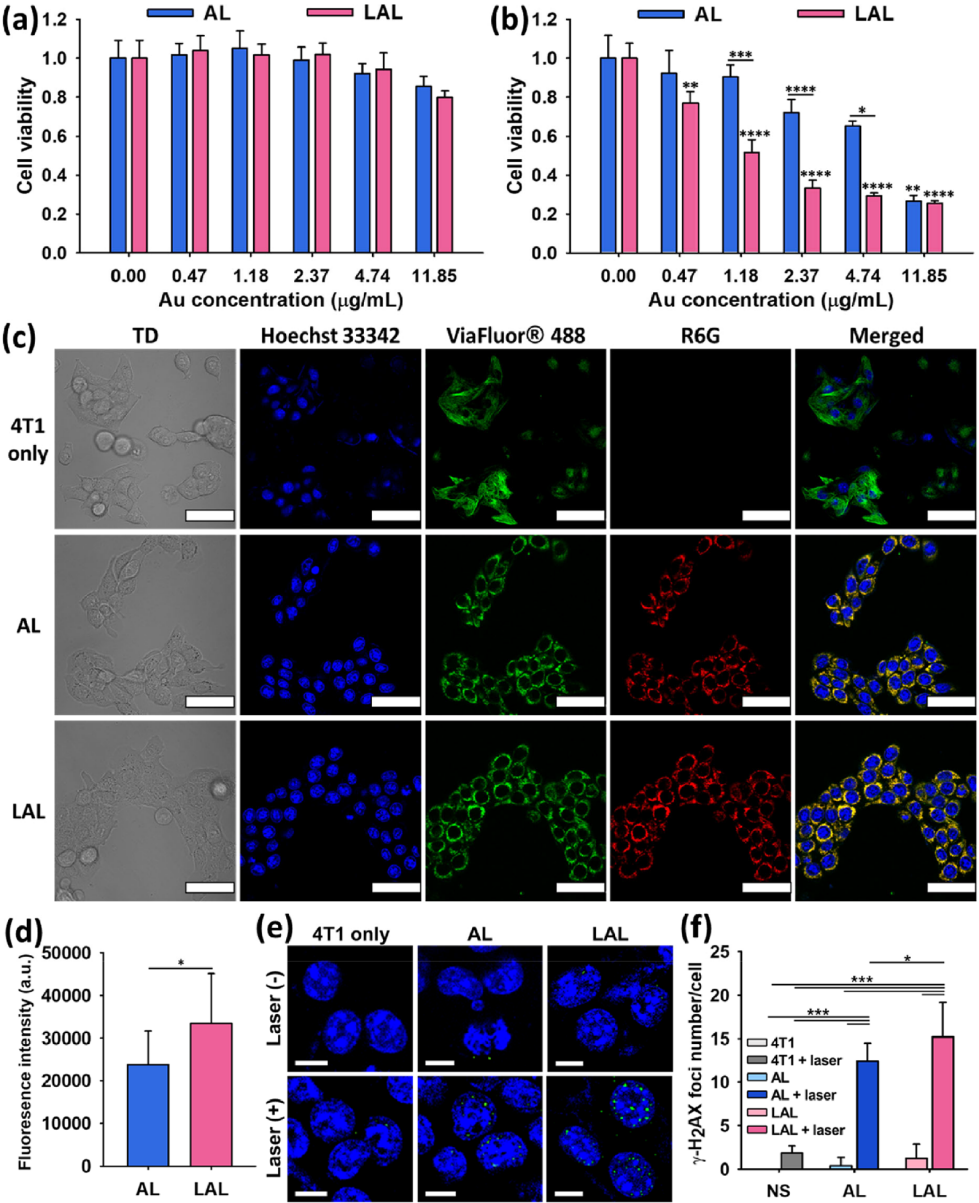
In vitro PTT results of AL and LAL. Cell viability of 4T1 treated AL and LAL **a** without laser and **b** with the 2 W cm^−2^ laser irradiation for 5 min (n = 4, mean ± s.d.). *P < 0.05, **P < 0.01, ***P < 0.001, ****P < 0.0001. Data were analyzed by one-way ANOVA with Tukey’s post-test. **c** Confocal images of AL and LAL internalized in 4T1 cells. All scale bars in the images are 50 μm. TD: transmitted light channel, blue: nuclei (Hoechst 33342), green: cytoskeleton (ViaFlour^®^ 488), red: LAL or AL (R6G). **d** The quantified fluorescence intensity of each cell where AL or LAL internalized (n = 14, mean ± s.d.). Data were analyzed by Student’s t-test. *P < 0.05. **e** The fluorescence images representing 4T1 cell nuclei to observe DNA-DSBs by the γ-H2AX foci after the laser irradiation (808-nm laser, 2.5 W cm^−2^, 5 min). Blue: nuclei (Hoechst 33342), green: γ-H2AX focus (Alexa Flour 488). All scale bars in the images are 10 μm. **f** DNA-DBSs quantitative analysis in 4T1 cells with and without laser irradiation (n = 5, mean ± s.d.). *P < 0.05, ***P < 0.001. Data were analyzed by one-way ANOVA with Tukey’s post-test

The cytotoxicities of LAL and AL were assessed (Fig. 3a). AL and LAL showed no overt cytotoxicity showing over 80% survival of the cells up to Au concentrations of 11.85 μg mL^−1^. In previous studies that utilized Au NP for cancer therapeutics, the concentration of Au was ranged from 15 to 200 μg per mouse (about 20 g) [42–48]. Therefore, both AL and LAL are reasonably biocompatible compare to previously reported studies. In vitro PTT effects of LAL and AL were compared in a triple-negative breast cancer cell line, 4T1 cell line, under the NIR irradiation. After the NIR laser irradiation to LAL incubated cells, tumor cell death was observed even at a low Au concentration of 0.47 μg mL^−1^ (Fig. 3b). Also, under the laser irradiation, LAL had higher cancer cell killing effect than the AL at all Au concentrations (from 0.47 to 4.74 μg mL^−1^) except the highest concentration, 11.85 μg mL^−1^. The in vitro PTT effects were significantly higher in LAL than AL at Au concentration of 1.18, 2.37, and 4.74 μg mL^−1^ (Fig. 3b) (P = 0.000745, P = 0.0000552, and P = 0.0162, respectively).

We explored the potential reasons for the significant difference of in vitro PTT effect between LAL and AL. Firstly, we compared the photothermal effect of AL and LAL in the physiological solution (RPMI 1640) to emulate in vitro situations (Additional file 1: Figure S4). After 40-min-irradiation, AL and LAL temperature changes were 21.4 ± 0.23 °C and 24.2 ± 1.65 °C, respectively. The temperature change of LAL was considerably higher than AL (P = 0.045). Because the efficient cell uptake of NP helps obtain maximum PTT effect, the cell uptake ability was compared using rhodamine 6G (R6G) loaded AL and LAL [49]. Cellular uptake of AL and LAL was observed in the red fluorescence images (Fig. 3c). We found that the fluorescence signal of the internalized LAL was significantly higher than that of internalized AL (P = 0.0163) (Fig. 3d). Finally, we compared the degree of DNA damage between AL and LAL based PTT. The PTT caused DNA damages were observed using a DNA-double strand breaks (DSBs) marker, γ-H2AX foci. DNA-DSBs of 4T1 cells were rarely detected in the 4T1, 4T1 + laser, AL, and LAL groups (Fig. 3e). AL + laser and LAL + laser exhibited much severe DNA damages in cells than the laser non-irradiated AL and LAL (P < 0.001). The number of γ-H2AX foci tended to be higher in the LAL + laser cells than the AL + laser cells (P < 0.05) (Fig. 3f).

Taken together, we found that LAL and AL both had excellent in vitro PTT effects and the PTT effect of LAL was higher than AL. The higher in vitro PTT effect of LAL on 4T1 cells than AL could be attributed to the higher cellular uptake, DNA damage, and photothermal effect in the physiological solution of LAL than AL.

### In vivo fluorescence imaging and quantitative analysis

**Fig. 4:**
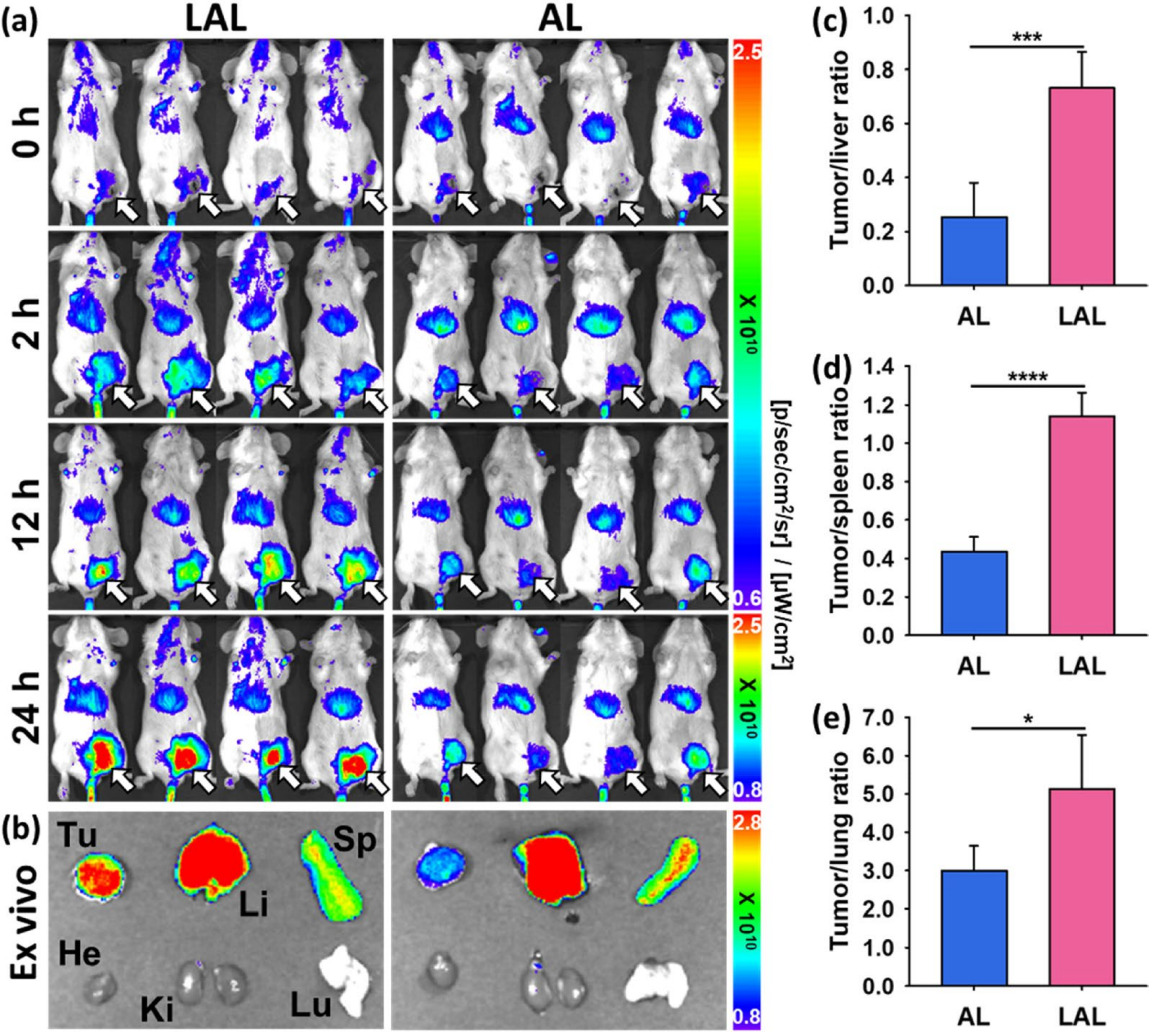
In vivo fluorescence images and assessment of passive tumor targeting efficacy of DiR labeled LAL and AL in 4T1 tumor bearing mouse model (n = 4). **a** In vivo fluorescence images of 4T1 breast cancer bearing mouse model at 0, 2, 12, and 24 h. DiR labeled LAL (left column) and AL (right column) injected mice. White arrows indicate tumors. **b** Representative ex vivo fluorescence images of tumors and main organs (He: heart, Li: liver, Sp: spleen, Ki: kidney, and Lu: lung) resected from each mouse after in vivo imaging. Comparison of tumor to non-targeted organ, **c** liver, **d** spleen, and **e** lung ratio. Tumor targeting efficiency comparison with non-targeted organ, **c** liver, **d** spleen, and **e** lung. Data were analyzed statistically with a Student’s t-test. *P < 0.05, ***P < 0.001, ****P < 0.0001

We assessed the tumor targeting abilities of AL and LAL in the 4T1 orthotopic breast cancer mouse model using in vivo fluorescence imaging. The same 20 μg of Au was injected to each mouse. For the in vivo fluorescence imaging, fluorescent AL and LAL were prepared using 1,1ʹ-dioctadecyl-3,3,3ʹ,3ʹ-tetramethylindotricarbocyanine iodide (DiR) labeled inner liposome. The imaging was performed up to 24 h after iv. injection of the NPs. DiR labeled LAL, and AL showed high liver and tumor uptakes. However, the tumor uptakes were more prominent in LAL injected mice than AL injected mice (Fig. 4a, b and Additional file 1: Figure S5). The mice were sacrificed after 24 h and the major organs and tumors were extracted. The fluorescence signals of the major organs and the tumors were quantified. Tumor to normal organ ratios were significantly higher in LAL than AL. The tumor to liver, spleen and lung ratios of LAL were 2.9, 2.6, and 1.7 folds higher than those of AL (Fig. 4c–e). We assumed that the additional liposomal layer of LAL enhanced the in vivo stability and caused the higher tumor uptake of the NPs.

### Radiolabeling efficiency and radiostability of ^64^Cu-LAL

**Fig. 5:**
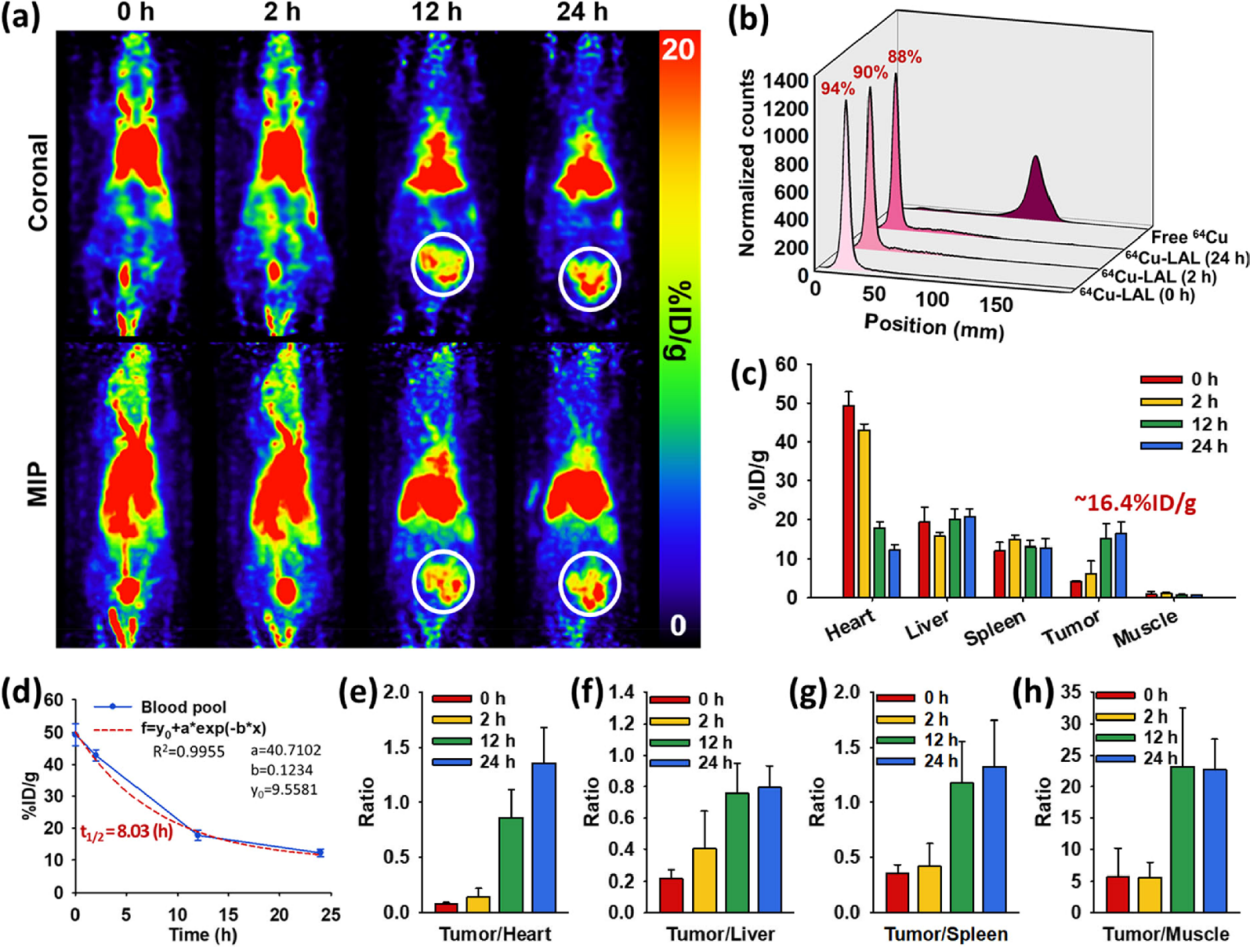
In vivo positron emission tomography (PET) images and assessment of passive tumor targeting efficiency of ^64^Cu-LAL. **a** Representative PET images of 4T1 breast cancer bearing mouse model (n = 3) at the different time points (0, 2, 12, and 24 h). White circles indicate tumor. Upper row: coronal, lower row: maximal intensity projection (MIP). **b** Radiochemical stability of ^64^Cu-LAL in PBS at 0, 2, and 24 h after radiolabeling. **c** Quantitative analysis of major organs (heart, liver, spleen, and muscle) and tumors from the PET images at each time point (n = 3, mean ± s.d.). **d** The circulation half-life from the time activity curve of blood pool. Tumor targeting efficiency comparison with non-targeted organ, **e** heart, **f** liver, **g** spleen, and **h** muscle (n = 3, mean ± s.d.)

A further modification cannot be applied easily in AL because of the decorated Au component on the liposome. In general, Au-thiol affinity was used for the functionalization of Au NPs, however, thiol moiety of Au-thiol can be easily replaced by glutathione (GSH) or other proteins [20, 32, 50, 51]. This leads to a detachment of radiolabeled-ligand, which causes uncertainty of Au in vivo biodistribution information. Thus, AL could not be radiolabeled and used in vivo with Au-thiol interaction. However, an additional lipid bilayer of LAL with 1,4,7-triazacyclononane-1,4,7-triacetic acid (NOTA) enabled further functionalization for radiolabeling with ^64^Cu. The radiolabeling efficiency of ^64^Cu-LAL in PBS was 94%, and the radiochemical stabilities were maintained at 90% and 88% after 2 and 24 h from the radiolabeling (Fig. 5b). With these results of high radiostability, we utilized ^64^Cu-LAL to evaluate the tumor targeting efficiency of LAL by in vivo PET imaging.

### In vivo PET imaging and quantitative in vivo biodistribution analysis

In vivo PET images were acquired to demonstrate the imaging ability of ^64^Cu-LAL (4.74 μg of Au) and assess the passive tumor targeting efficiency of the ^64^Cu-LAL in the orthotopic breast cancer mouse model (Fig. 5a). The PET images showed the long circulation ability and effective tumor targeting efficiency of ^64^Cu-LAL. The quantified ^64^Cu-LAL uptake of major organs in the PET images was shown in Fig. 5c. The ^64^Cu-LAL uptake in tumors increased gradually up to 16.4%ID g^−1^ after 24 h from the injection. The circulation half-life of ^64^Cu-LAL was calculated as 8.03 h (Fig. 5d). Tumor to background (heart, liver, spleen, and muscle) ratios exhibited an increasing tendency, and the ratios were 1.4, 0.79, 1.3, and 23, respectively, after 24 h from then injection (Fig. 5e–h). Of note, tumor to liver and tumor to spleen ratios based on PET images were similar with the results from IVIS imaging. In previous studies, various types of Au NPs for PTT were accumulated in tumors ranging from 1.61 to 9.6%ID g^−1^ after 12–48 h from the iv. injection [52–58]. Bare Au NPs showed the lowest tumor accumulation, 1.61%ID g^−1^. Most Au NPs were modified with PEG, peptides, or proteins, showing over 5%ID g^−1^ and the highest value, 9.6%ID g^−1^, was from PEGylated Au NPs (Table 1). Our ^64^Cu-LAL showed significantly higher accumulation (16.4%ID g^−1^) than previously reported Au NPs, which could be attributed to the double layers of liposome and successful PEGylation.

**Table 1:** Targeting efficacy of conventional Au-based NPs by inductively coupled plasma mass spectrometer (ICP-MS)

Particle name (commercial or by the authors)	Concentration of Au administered in vivo		Concentration of Au in tumor	Targeting (%ID/g)	Injection method	Refs
ELP-Au NPs		180 μg Au mL^−1^	0.15–0.18 μg mm^−3^	> 90 (after 1 day from injection) 34 (after 30 days from injection)	Intratumoral	[58]
GNPs		186 μg Au mL^−1^	~ 0.186 μg mm^−3^	~ 100 (right after injection) (this value was calculated from concentration of Au in tumor)	Intratumoral	[43]
sAuNR-laden-macrophages		2.1 μg Au μL^−1^	~ 0.525 μg mm^−3^	~ 100 (right after injection) (this value was calculated from concentration of Au in tumor)	Intratumoral	[47]
Bare AuNSs		3 mg mL^−1^	0.07245 μg mm^−3^	1.61 (after 48 h from injection)	Intravenous	[52]
MPCM-AuNSs		ND.	ND	7.48 (after 48 h from injection)	Intravenous	[52]
CPGA^a^		10.4 mg Au kg^−1^ (ND. of administrated volume)	~ 0.0147 μg mm^−3^	7.06 ± 0.49 (after 24 h from injection)	Intravenous	[56]
Au NPs		2 mg mL^−1^ (10 mg kg^−1^)	~ 0.02 μg mm^−3^ (after 12 h from injection)	1 (after 2 h from injection) 6 (after 12 h from injection) 2 (after 24 h from injection)	Intravenous	[54]
AuNP@peptide		ND	ND	3 (after 2 h from injection) 9 (after 12 h from injection) 7 (after 24 h from injection)	Intravenous	[54]

*ELP-Au NPs* elastin-like polypeptide (ELP) conjugated Au NPs, *GNPs* gold nanoparticle, *sAuNR* 7 nm diameter Au nanorods, *AuNSs* gold nanoshells, *MPCM-AuNS* macrophage cell membrane (MPCM)-camouflaged AuNS*, ND.*No data

^a^CPGA: near-infrared dye (Cy5.5) labeled-matrix metalloproteinase-14 (MMP-14) substrate (CP) was conjugated onto the GO/Au complex (GA) forming tumor targeted theranostic probe

Theranostics is a portmanteau word combining diagnostics and therapeutics, which referes to agents or techniques that couple diagnostic imaging with targeted therapy. The diagnostic imaging coupled with therapeutics is a valuable tool for precision medicine because it could stratify patietns who will respond to the therapeutics. We successfully radiolabled LAL and obtained in vivo PET imaging of LAL. Also we were able to quantify the tumor uptake and organ distribution using the PET images. This information could be used for deciding and optimizing dose for PTT.

### Effective in vivo PTT of LAL

To confirm that the 808-nm laser can occur a photothermal effect in vivo, we subcutaneously injected the NPs into normal BALB/c-nude mice and irradiated the laser. In both AL and LAL injected sites, increased temperature was observed, but the temperature change was significantly higher in LAL (40.0 °C) than AL (29.5 °C) (Additional file 1: Figure S6). This result corresponds with the in vitro comparison in physiological solution in Additional file 1: Figure S4, but the difference was more prominent in vivo. It has been reported that the aggregation of Au NPs reduces photothermal conversion efficiency [59]. Therefore, we assume that the instability of AL caused aggregation in vivo and resulted in a reduction of the in vivo photothermal effect.

**Scheme 2:**
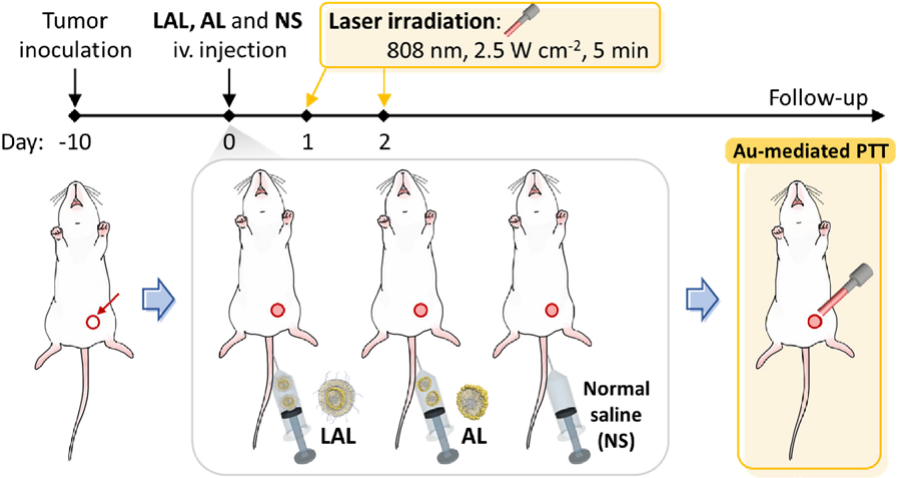
Schematic illustration of in vivo PTT in orthotopic 4T1 tumor bearing mice injected NS, AL, and LAL. 808-nm laser was irradiated on each tumor site with 2.5 W cm^−1^ intensity, and the laser irradiation procedure was performed twice after the iv. injection

**Fig. 6:**
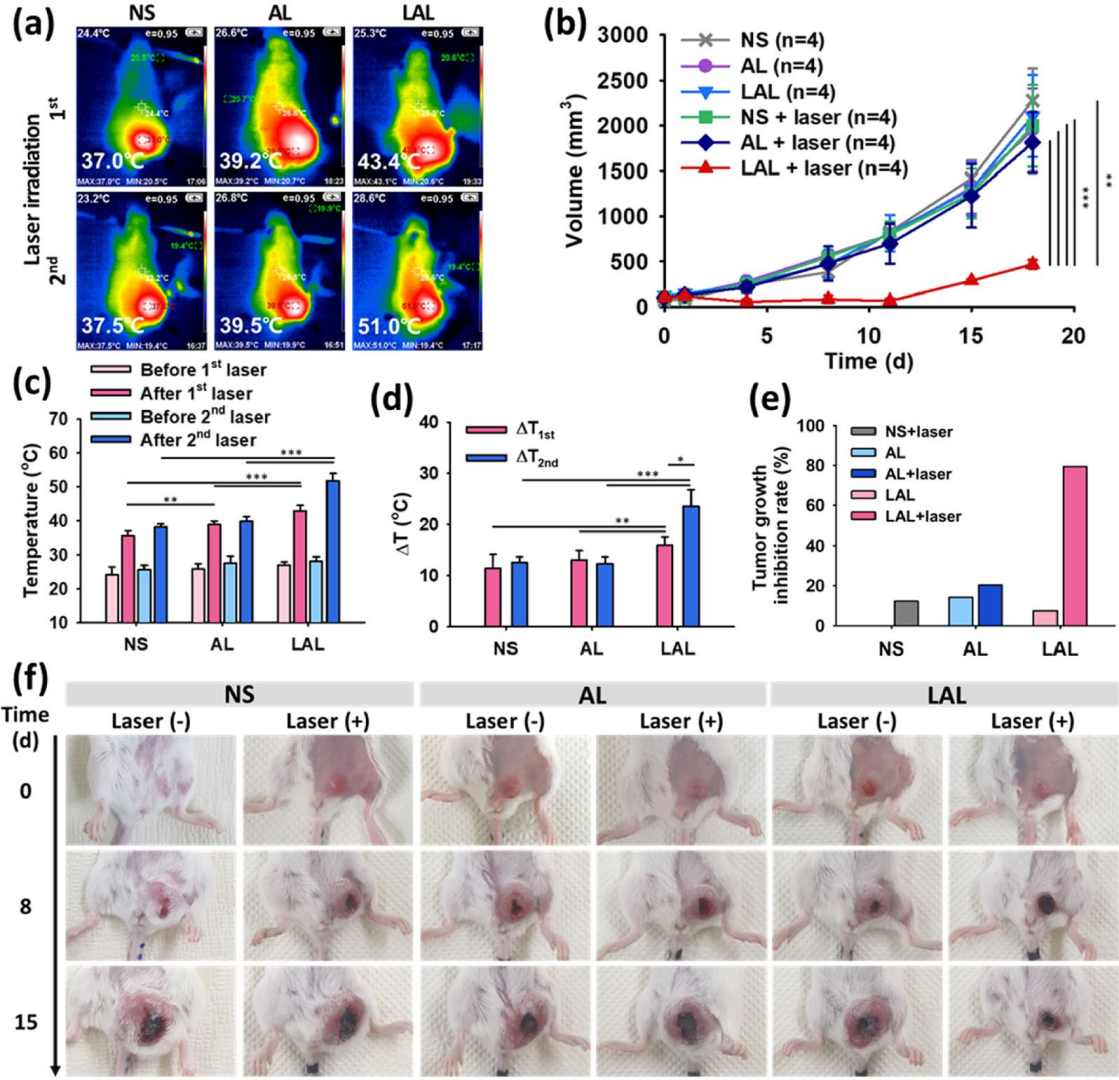
In vivo PTT results of 4T1 tumor bearing BALB/c mice treated with NS, AL, and LAL. **a** Representative thermal images of 4T1 breast cancer bearing mouse models while the laser irradiated (808-nm, 2.5 W cm^−2^, 5 min). (Upper row: 1st irradiation (24 h after the injection), lower row: 2nd irradiation (48 h after the injection)). **b** Tumor volumes of 4T1 tumor bearing BALB/c mice treated with NS, AL, and LAL. Each treatment group was divided into two groups, with or without laser irradiation. (n = 4, mean ± s.d.). Tumor size was measured at 0, 1, 4, 8, 11, 15, and 18 days. **c** Temperature elevation and **d** the temperature changes of NS, AL, and LAL under the laser irradiation (n = 4, mean ± s.d.). Data were analyzed by one-way ANOVA with Tukey’s post-test. ** is P < 0.01 and *** is P < 0.001. **e** In vivo tumor growth inhibition rate of 4T1 tumor bearing BALB/c mice after the PTT with NS, AL, and LAL. **f** Representative photographs of 4T1 tumor bearing BALB/c mice exhibiting tumor growth at 0, 8, and 15 days

After validating the above results, we conducted further experiments to examine the efficacy of PTT in vivo with 4T1 tumor bearing mice by iv. injection with AL and LAL (of note, 20 μg of Au per mouse was used in both AL and LAL). We divided the mice into six groups for the comparison: normal saline (NS) without laser irradiation, NS with laser irradiation, AL without laser irradiation, AL with laser irradiation, LAL without laser irradiation, and LAL with laser irradiation (NS, NS + laser, AL, AL + laser, LAL, and LAL + laser) (Scheme 2). Since the tumor uptake is almost plateaued 24 h after the LAL injection based on the PET in vivo biodistribution data, the first laser irradiation was given 24 h after the injection. The second laser irradiation was carried out 24 h later from the first laser irradiation. While irradiating the 808-nm laser, thermal images were obtained (Fig. 6a, Additional file 1: Figure S7). The temperature of LAL groups prominently elevated to 43.4 °C at the first irradiation and 51.0 °C at the second irradiation, while NS and AL groups showed a mild elevation of the temperature [37 °C (1st) and 37.5 °C (2nd) in NS group; 39.2 °C (1st) and 39.5 °C (2nd) in AL group, respectively] (Fig. 6c). As we verified the thermal stabilities of both LAL and AL, we also demonstrated the temperature elevation of tumor sites, which were laser-irradiated. The temperature changes of the LAL group were significantly higher than those of NS and AL groups on both irradiation (1st irradiation, P < 0.01; 2nd irradiation, P < 0.001) (Fig. 6d). These substantial temperature changes in LAL group induced feverish environments to tumor tissues which could provoke tumor cell killing effect through the hyperthermia mechanism.

As shown in Fig. 6b, f, and Additional file 1: Figure S8, only the LAL with the laser irradiation group showed effectively suppressed tumor growth among the six groups. The AL, LAL, NS + laser, and AL + laser groups showed similar tumor growth with the control NS group. The tumor growth inhibition rate is defined following equation, (1 − (mean volume of treated tumors)/(mean volume of control tumors)) × 100% [60]. With this definition, the tumor growth inhibition rate of each group is as follows: NS + laser was 12.2%, AL was 14.2%, AL + laser was 20.4%, LAL was 7.52%, and LAL + laser was 79.4% (Fig. 6e). Both tumor volume and tumor growth inhibition rate showed that LAL with PTT was far more effective than AL with PTT. LAL + laser group also showed a significantly higher therapeutic effect in tumor sectioned images (Additional file 1: Figure S9). Furthermore, we assessed the in vivo toxicity of the NPs by observation of the histology of major organs after the PTT and found that there were no visible damages in the heart, liver, spleen, kidney, and muscle (Additional file 1: Figure S9). Taken together, LAL was more effective at inhibiting tumor growth than AL. This is because LAL has improved stability compared to AL, resulting in sustained photothermal effects in vivo and increased tumor passive targeting efficiency. As the functionalized outer liposomal layer stabilized LAL in in vivo environment, enhanced permeability and retention (EPR) effect of LAL could be remarkably increased. Photothermal effect was also higher in the LAL treated mice, since LAL were concentrated in the tumor much more than AL. Consequently, more efficient anti-tumor effect was observed in PTT with LAL.

LAL showed high tumor targeting in both fluorescence images and PET images with its passive targeting ability only. The delivery efficacy of LAL, nevertheless LAL has no specific targeting moiety, is in the top 8.6% (21st) among the previously reported NPs according to the review paper (Additional file 1: Figure S10) [61]; the review covers NPs with passive or active targeting ability. As a one way to maximize PTT effect with LAL, approaches for active tumor targeting could be considered by adding target ligands, aptamers, peptides, and antibodies [62–64]. A single domain antibody is one of the active targeting moieties and have advantages of high physicochemical stability, rapid tissue penetration, and facile genetic manipulation [65]. Single domain antibodies have been recently used in liposome research which is limited in vitro experiments [66–68]. Attaching the active targeting moiety to the LAL, which already showed the high passive tumor targeting, it is expected to have a synergistic tumor targeting efficiency.

Another way to increase the PTT effect is using immune checkpoint inhibitors (ICI). Recently, NP based PTT is attracting more attention as a promising anti-tumor strategy because of its ability to enhance the effect of ICI [69]. The major mechanism to enhance the effect of ICI by PTT is to convert immunologically “cold” tumor into “hot” tumor, which responds better to ICI. Multiple PTT studies using different PTT agents showed the synergistic effect with ICI. Indocyanine green (ICG), a photothermal agent, loaded poly(lactic-*co*-glycolic) acid (PLGA) NP demonstrated improved efficacy of checkpoint-blockade using anti-cytotoxic T-lymphocyte antigen-4 (anti-CTLA4) [70]. Huang et al. reported that a lipid gel depot loaded with IR820 (PTT agent) and anti programmed death-ligand 1 (PD-L1) antibody (ICI) could induce increased lymphocyte infiltration and anti-tumor activity in “cold” tumors [71]. Lu et al. reported that polydopamine based NP mediated PTT was able to enhance anti PD-L1 therapy by activating both innate and adaptive immune systems [72]. We also expect LAL could be further utilized for enhancement of ICI based on its excellent tumor targeting ability and PTT effect.

## Conclusions

We developed LAL by covering AL with additional liposome layer for the effective in vivo imaging and target-specific PTT. LAL showed better stability, tumor targeting, and in vitro/in vivo PTT effect than AL. We found that LAL has (1) high photothermal conversion efficiency, (2) high in vivo stability and passive targeting efficiency, and (3) excellent in vitro/in vivo PTT effect. Also, the passive targeting ability of LAL outperformed previously reported Au based PTT agents. Therefore, LAL could be a promising Au based PTT agent that can be injected intravenously.

## Methods

### Materials

Distearoyl phosphatidylcholine (DSPC) was purchased from Avanti Polar Lipids, Inc. (Alabaster, AL, USA). 1,2-Distearoyl-sn-glycero-3-phosphoethanolamine (methoxy(polyethylene glycol)-5000) (DSPE-PEG(5k)) and 1,2-Distearoyl-sn-glycero-3-phosphoethanolamine-*N*-[amino(polyethylene glycol)-2000] (DSPE-PEG(2K)-NH_2_ were obtained from Creative PEGworks (Chapel Hill, NC, USA). Cholesterol (chol), l-ascorbic acid, citric acid, sodium acetate, thiazolyl blue tetrazolium bromide, and Roswell Park Memorial Institute (RPMI) 1640, Triton X-100, and bovine serum albumin were purchased from Sigma-Aldrich (St. Louis, MO, USA). Hydrogen tetrachloroaurate tetrahydrate (HAuCl_4_∙4H_2_O) was acquired from Kojima Chemicals (Saitama, Japan). 2-(*p*-Isothiocyanatobenzyl)-1,4,7-triazacyclononane-*N*,*N*ʹ,*N*ʺ-triacetic acid trihydrochloride (*p*-SCN-Bn)-NOTA (> 95%) was purchased from FutureChem Co., Ltd. (Seoul, Korea). Size exclusion PD-10 column and fetal bovine serum (FBS) were obtained from GE Healthcare Life Science (Buckinghamshire, UK). Radio-instant thin layer chromatography silica gel (ITLC-SG) was acquired from Agilent Technologies, Inc. (Santa Clara, CA, USA). 4% paraformaldehyde (PFA) was purchased from Biosesang (Seongnam, Korea). Phospho-Histone H2A.X (Ser140) monoclonal antibody (3F2) and 1,1ʹ-dioctadecyl-3,3,3ʹ,3ʹ-tetramethylindotricarbocyanine iodide (DiR) were purchased from Invitrogen (Carlsbad, CA, USA). Goat Anti-Mouse IgG H&L (Alexa Fluor 488) was acquired from Abcam (Cambridge, UK). ViaFluor^®^ 488 Live Cell Microtubule Staining Kit was acquired from Biotuim (Fremont, CA, USA). Female BALB/c and BALB/c nude mice (6–8 weeks) were obtained from Orient Bio (Seongnam, Korea).

### Instruments

All hydrodynamic sizes and zeta potential values were characterized by dynamic light scattering (DLS, Zetasizer Nano-ZS, Malvern Instrument Ltd., Worcestershire, UK). Energy-Filtering Transmission Electron Microscope (EF-TEM, 120 kV, LIBRA 120, Carl Zeiss, Oberkochen, Germany) was used to confirm the nanostructures. Absorbance and fluorescence were measured by a microplate reader (SYNERGY H1, BioTek, Winooski, VT, USA). To conduct a photothermal effect and PTT experiments, 808-nm NIR laser (FC-W-808-10W, CNI, Changchun, China) and thermal imaging camera (HT-18, HT instrument, Faenza, Italy) were used. A confocal microscope (Nikon A1R, Nikon Co., Tokyo, Japan) was operated to observe cellular uptake. In vivo mice fluorescence images were obtained using in vivo imaging system (IVIS, IVIS Lumina X5 Imaging System, Perkin-Elmer, Waltham, MA, U.S). Animal PET (GENISYS, Sofie Biosciences, Culver City, CA, USA) was used to observein vivo biodistribution images and the images were analyzed by MIMvista (MIM Software Inc.).

### Preparation of liposome-coated Au-liposome (LAL)

2.5:1 molar ratio of DSPC and cholesterol were dissolved in the mixture of chloroform and methanol. The mixture was dried completely, and the pre-liposomal lipid film was formed. The lipid film was hydrated with deionized water (DW) and dispersed by forming multilamellar vesicles. The liposomal solution was prepared after ultrasonication, and the liposome was purified with the 0.2-μm syringe filter and size exclusion chromatography with a PD-10 column. The prepared inner liposome was decorated with Au using a 1% (wt/v) HAuCl_4_∙4H_2_O solution and a 10% (wt/v) ascorbic acid solution, which was Au-liposome (AL). This process occurred with a color change from the translucent white liposomal solution to the bluish-green color solution. The outer liposomal layer with DSPC:chol:DSPE-PEG(5k) = 2.5:1:0.3 molar ratio was prepared by the same procedure as the inner liposome. The only difference is it was hydrated with AL suspension, not DW. Afterward, liposome coated Au-liposome (LAL) was purified by a syringe filter and a PD-10 column. Hydrodynamic sizes of AL and LAL were characterized by DLS, and TEM images were obtained after negative stain with a 2% uranyl acetate solution. Every zeta potential was measured in 5 mM phosphate buffer (pH 7.4) and repeated 12 times.

### In vitro stability

The prepared AL and LAL were diluted by Au concentration 4.74 μg mL^−1^ in DW, phosphate-buffered saline (PBS), and RPMI 1640 medium containing 10% FBS and 1% penicillin/streptomycin to demonstrate the stabilities in physiological conditions for 14 days at room temperature. Hydrodynamic sizes were measured at every time point by DLS.

### In vitro photothermal effect and photothermal efficiency

Before demonstrating the photothermal effect, the absorbance spectra of liposome, AL, and LAL were measured by a microplate reader to confirm that AL and LAL absorbed 808-nm light. In vitro photothermal effect and efficiency of AL and LAL were demonstrated by the 808-nm laser with 1 W intensity. Using a thermal imaging camera, the temperature changes of DW, liposome, AL, and LAL under the laser irradiation were measured for 40 min, and the thermal images were obtained simultaneously. The thermal stabilities of AL and LAL demonstrated by the on/off cycle of laser irradiation for 20 min and cooling down for 10 min. This cycle was repeated 4 times, and the temperatures were measured every 30-s.

The photothermal conversion efficiency (η value) was calculated with Eq. 1, above mentioned.

1 $$\eta =\frac{\mathrm{h}A\left({T}_{\mathrm{max}}-{T}_{\mathrm{sur}}\right)-{Q}_{\mathrm{diss}}}{I(1-{10}^{-A})}=\frac{\mathrm{mc}\left({T}_{\mathrm{max}}-{T}_{\mathrm{sur}}\right)-{Q}_{\mathrm{diss}}}{{\tau }_{s}I(1-{10}^{-A})}$$

The heat conversion was investigated by irradiating the AL and LAL (Au 23.7 μg mL^−1^) in DW with the 1 W laser irradiation for 20 min. *T*_max_ is the highest temperature of NP solution, and *T*_sur_ is the initial temperature of NP solution. *Q*_*diss*_ is the heat dissipation [73, 74], and $I$ represents the power of laser. *A* is the absorbance at 808 nm, *m* is the weight of NP solution, and *c* represents the specific heat capacity of water. *τ*_s_ was determined by Eq. 2.

2 $$\tau_{{\text{s}}} { } = - \frac{t}{ln\theta }$$

*θ* refers to the dimensionless driving force, and *t* represents the corresponding time.

### Cytotoxicity test and in vitro PTT

4T1 breast cancer cells were cultured at 2 × 10^4^ cells per well in 96-well plates and incubated overnight at 37 °C under 5% CO_2_. The RPMI 1640 medium containing 10% FBS and 1% penicillin/streptomycin was used for the experiment. AL and LAL were added to each well with various concentrations from 0 to 11.85 μg mL^−1^. Then, the cells were incubated for 24 h. Cells were washed with DPBS to remove AL and LAL, and then the MTT solution was treated to the cells. Cell viability was demonstrated by measuring the absorbance at 540 nm. To demonstrate in vitro PTT, the 808-nm laser was irradiated to the cells with 2.5 W cm^−2^ for 5 min after 4 h from the AL and LAL addition. The other procedures were the same without the laser irradiation cytotoxicity test.

### Cellular uptake of AL and LAL imaging

R6G was loaded in the innermost liposome to observe cellular uptake of AL and LAL. The R6G loaded liposome was filtered using both a syringe filter and PD-10 column. 4T1 cells were incubated overnight in confocal dishes at 37 °C under 5% CO_2_. Both AL and LAL (Au 9.48 μg mL^−1^) were added to the cells, and the cells were incubated for 4 h and then washed the AL and LAL. Hoechst 33342 and ViaFluor^®^ 488 were added for staining nuclei and cytoskeleton of the cells, and the cells were washed 3 times. The cellular uptake of each AL and LAL were observed by confocal microscopy. The corrected total cell fluorescence (CTCF) was calculated from the fluorescence images using the free software, ImageJ (n = 14).

### Immunofluorescence

7 × 10^4^ 4T1 breast cancer cells were incubated in confocal dishes overnight at 37 °C under 5% CO_2_. Cells were treated with AL and LAL for 4 h and irradiated the 2.5 W cm^−2^ of 808-nm laser for 5 min. After 1 h from the laser irradiation, cell nuclei were stained with Hoechst 33342 and then fixed with 4% paraformaldehyde at 37 °C for 10 min. Cells were permeabilized in 0.2% Triton X-100 for 10 min and then treated with 3% BSA in PBS for 30 min. Phospho-Histone γ-H2A.X monoclonal primary antibody was added and incubated overnight at 4 °C. After washing with PBS, cells were also added goat anti-mouse IgG H&L (Alexa Fluor 488) secondary antibody in 3% BSA for 30 min. Images of γ-H2AX foci and nuclei were obtained by confocal microscopy. The number of γ-H2AX foci per cell was quantified with ImageJ (n = 5).

### Preparation of orthotopic 4T1 breast cancer model

4T1 breast cancer bearing mice were prepared for in vivo PET imaging, in vivo mice fluorescence imaging, photothermal effect, and PTT. 4T1 cell line (10^5^ cells 15 μL^−1^ of PBS) was injected into the left fifth nipple. In vivo imaging and PTT experiment with 4T1 tumor bearing mice was performed when the tumor volumes were 150–300 mm^3^ and 50–100 mm^3^. All the animal experiments were approved by Institutional Animal Care and Use Committee at Seoul National University.

### In vivo fluorescence imaging with DiR labeled AL and LAL

DiR was added when the inner liposome was prepared. DiR labeled inner liposome was purified by a syringe filter and a PD-10 column. The next procedures to prepare DiR labeled AL and LAL were the same above mentioned. DiR labeled AL and LAL (Au 20 μg) were injected into the 4T1 breast cancer bearing mice (n = 4) via tail vein. In vivo images were acquired at 0, 2, 12, and 24 h from the injection, and the mice were sacrificed. Ex vivo fluorescence images of the tumors and main organs were acquired. Each tumor to liver, spleen, and lung ratio were analyzed statistically with a Student’s t-test, two-sided.

### Radiolabeling efficiency and radiostability of LAL

For radiolabeling of LAL, the outer liposomal layer of LAL was composed of distearoyl phosphatidylcholine (DSPC), cholesterol, 1,2-Distearoyl-sn-glycero-3-phosphoethanolamine (methoxy(polyethylene glycol)-5000) (DSPE-PEG(5k)), and 1,4,7-triazacyclononane-1,4,7-triacetic acid (NOTA) modified 1,2-Distearoyl-sn-glycero-3-phosphoethanolamine-*N*-[amino(polyethylene glycol)-2000] (DSPE-PEG(2K)). The (p-SCN-Bn)-NOTA was reacted with DSPE-PEG(2K)-NH_2_ overnight, and the prepared NOTA modified DSPE-PEG(2K) was added to the outer pre-liposomal lipid mixture. The following procedure was the same as the LAL procedure mentioned above. NOTA modified LAL and ^64^Cu (II) ion were reacted in pH 5 sodium acetate buffer solution at 37 °C for an hour. Size exclusion chromatography was conducted to eliminate the free ^64^Cu (II) ion.

To demonstrate radiostability, ^64^Cu labeled LAL (^64^Cu-LAL, 2 μL) and ^64^CuCl_2_ solution was loaded onto the ITLC-SG plate, and thin-layer chromatography (TLC) was conducted with a 0.1 M citric acid solution after 0 to 24 h from a size exclusion chromatography. Radiolabeling efficiency of ^64^Cu-LAL at each time point was measured based on the TLC result.

### In vivo PET imaging and quantitative in vivo biodistribution analysis

^64^Cu-LAL (200 μL, Au 4.74 μg) was injected intravenously into every mouse (n = 3) anesthetized with 2% isoflurane to confirm the in vivo biodistribution data and tumor targeting efficiency. PET images were acquired with an animal PET instrument. The three-dimensional region of interest (ROI) values of major organs (including heart, liver, spleen, and muscle) and tumor were analyzed by MIM software.

### In vivo photothermal effect

To confirm the in vivo photothermal effect, LAL and AL with 2.37 μg of Au (50 μL) were injected into normal BALB/c nude mice subcutaneously. The laser was irradiated to the AL and LAL injected site of each mouse (n = 3) anesthetized with 2% isoflurane with 2.5 W cm^−2^ laser intensities for 5 min. Each temperature was measured by a thermal imaging camera before and after the laser irradiation.

### In vivo PTT

Prepared LAL, AL, and NS were injected into the 4T1 breast cancer bearing mice via tail vein. The same amount of Au (20 μg) was loaded in AL and LAL. The 808-nm laser was directly irradiated to the tumor site with 2.5 W cm^−2^ intensities, and the laser irradiation procedures were carried out twice in this experiment, and the mice were anesthetized with 2% isoflurane. The first laser irradiation was conducted after 24 h from the iv. injection and the second laser irradiation was conducted after 48 h from the iv. injection. Thus, in this in vivo PTT experiment, there were 6 groups (n = 4) as follows: LAL without laser irradiation, LAL with laser irradiation, AL without laser irradiation, AL with laser irradiation, NS without laser irradiation, and NS with laser irradiation (NS, NS + laser, AL, AL + laser, LAL, and LAL + laser). Photothermal images were obtained simultaneously irradiating the laser, and the acquired temperatures were compared by one-way ANOVA with Tukey’s post-test. Tumor sizes were measured up to 18 days from the LAL, AL, and NS injection, and tumor images were obtained on the same day. Tumor volumes were calculated using the following formula,

$${\text{V}}\, = \,{3}/{4}\, \times \,\pi \, \times \,{\text{a}}/{2}\, \times \,\left( {{\text{b}}/{2}} \right)^{{2}} ,$$

where a and b are the larger and smaller diameters, respectively. After the PTT, major organs (heart, liver, spleen, kidney, lung, muscle, and intestine) and tumor in each group were paraffin sectioned, and H&E stained for the tissue imaging.

## Supplementary Information

**Additional file 1.** Additional figures (**Figure S1**–**S10**).

## Data Availability

All data generated or analyzed during this study are included in this published article.

## Abbreviations

PTT: Photothermal therapy
Au: Gold
AL: Gold coated liposome
LAL: Liposomal coated AL
PEG: Polyethylene glycol
PET: Positron emission tomography
NIR: Near-infrared
Au NPs: Gold nanoparticles
RES: Reticuloendothelial system
iv.: Intravenous
TEM: Transmission electron microscopy
DNA-DSBs: DNA-double strand breaks (DSBs)
DiR: 1,1ʹ-Dioctadecyl-3,3,3ʹ,3ʹ-tetramethylindotricarbocyanine iodide
He: Heart
Li: Liver
Sp: Spleen
Ki: Kidney
Lu: Lung
GSH: Glutathione
MIP: Maximal intensity projection
ICP-MS: Inductively coupled plasma mass spectrometer
NS: Normal saline
EPR: Enhanced permeability and retention
H&:E: Hematoxylin and eosin
ICI: Immune checkpoint inhibitor
ICG: Indocyanine green
PLGA: Poly(lactic-*co*-glycolic) acid
CTLA4: Cytotoxic T-lymphocyte antigen-4
PD-L1: Programmed death-ligand 1
