# Supplementary material for: Development of theranostic dual-layered Au-liposome for effective tumor targeting and photothermal therapy
Source: J Nanobiotechnology. 2021 Sep 4;19:262. doi: 10.1186/s12951-021-01010-3 (PMC8418714; doi:10.1186/s12951-021-01010-3)
Supplement: Supplementary file 1 — Additional file 1. Additional figures (Figure S1–S10). [file 12951_2021_1010_MOESM1_ESM.docx]

Additional Information

**Development of theranostic dual-layered Au-liposome for effective tumor targeting and photothermal therapy**

Miyeon Jeon, Gaeun Kim, Wooseung Lee, Seungki Baek, Han Na Jung, Hyung-Jun Im*

**Figure S1.** Stability test of AL and LAL in the different physiological conditions. Stability under DW, PBS, and cell media (RPMI 1640 with 10% FBS). Only AL exhibited visible aggregation over time, while LAL showed little differences. Right: DW, middle: PBS, and Left: RPMI 1640.


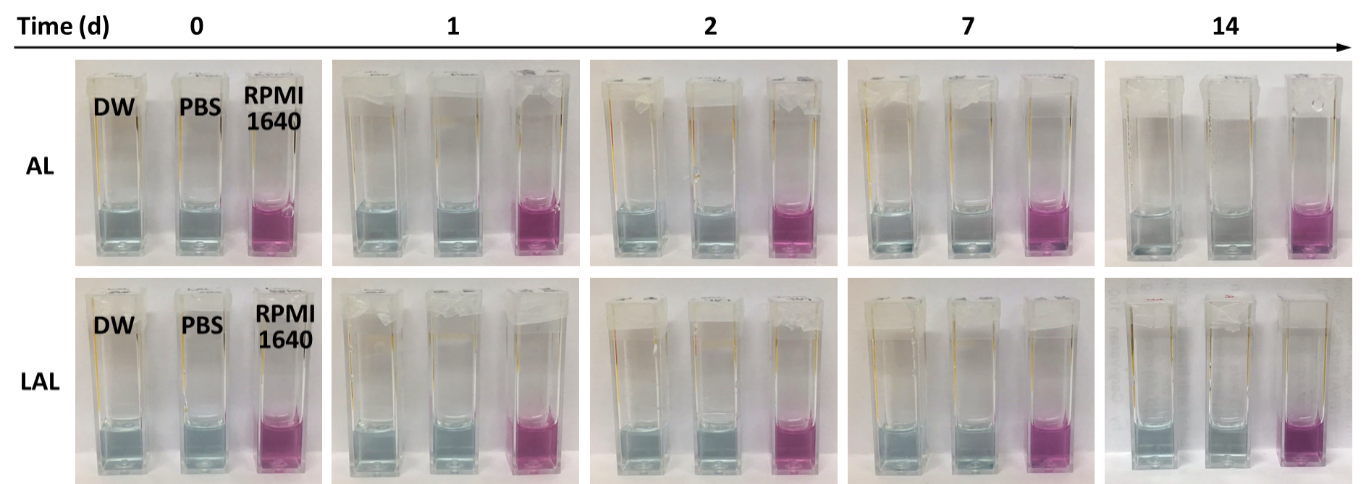


**Figure S2.** Hydrodynamic size variations of LAL and AL over time to identify the stability. Hydrodynamic sizes of (a) LAL and (b) AL during 2 weeks in DW, PBS, and RPMI 1640 with 10% FBS.

**
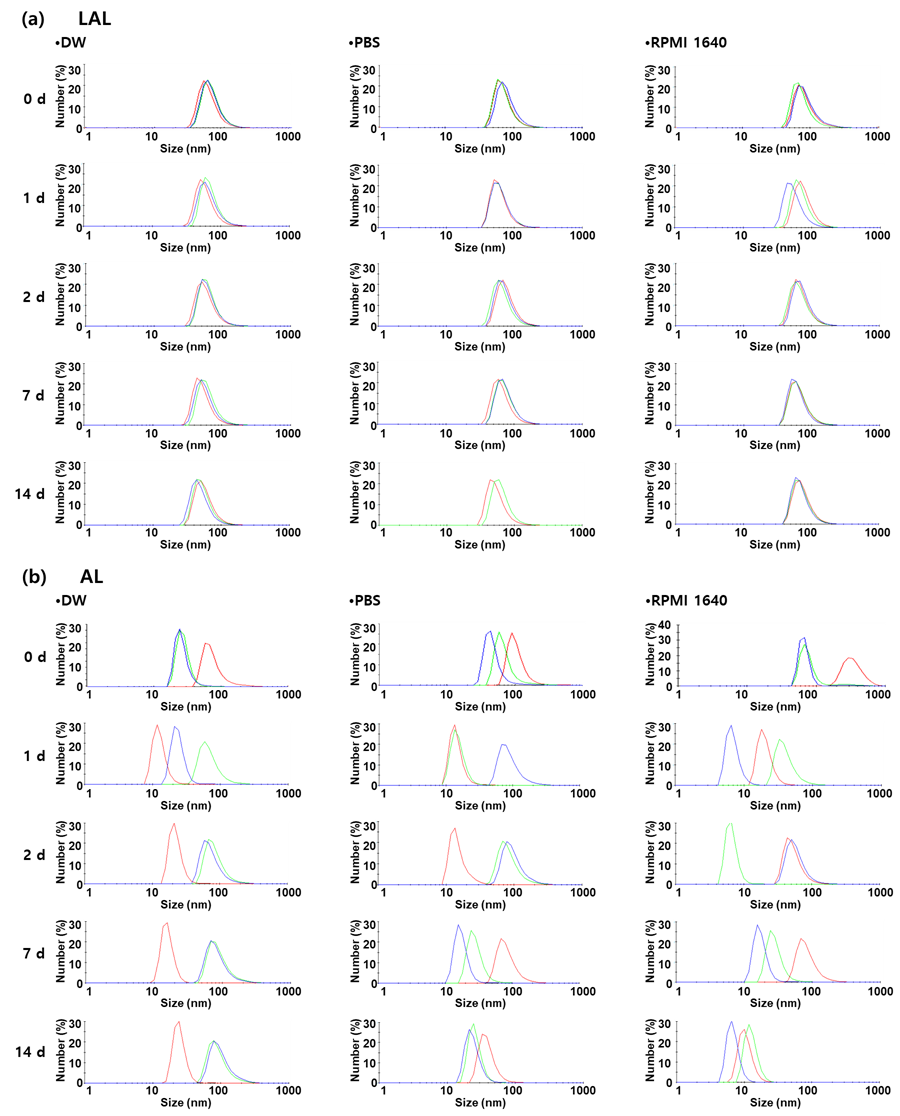
**

**Figure S3.** Temperature elevation of LAL, AL, liposome and DW under the 808-nm laser irradiation with 1 W intensity for 40 minutes.

**
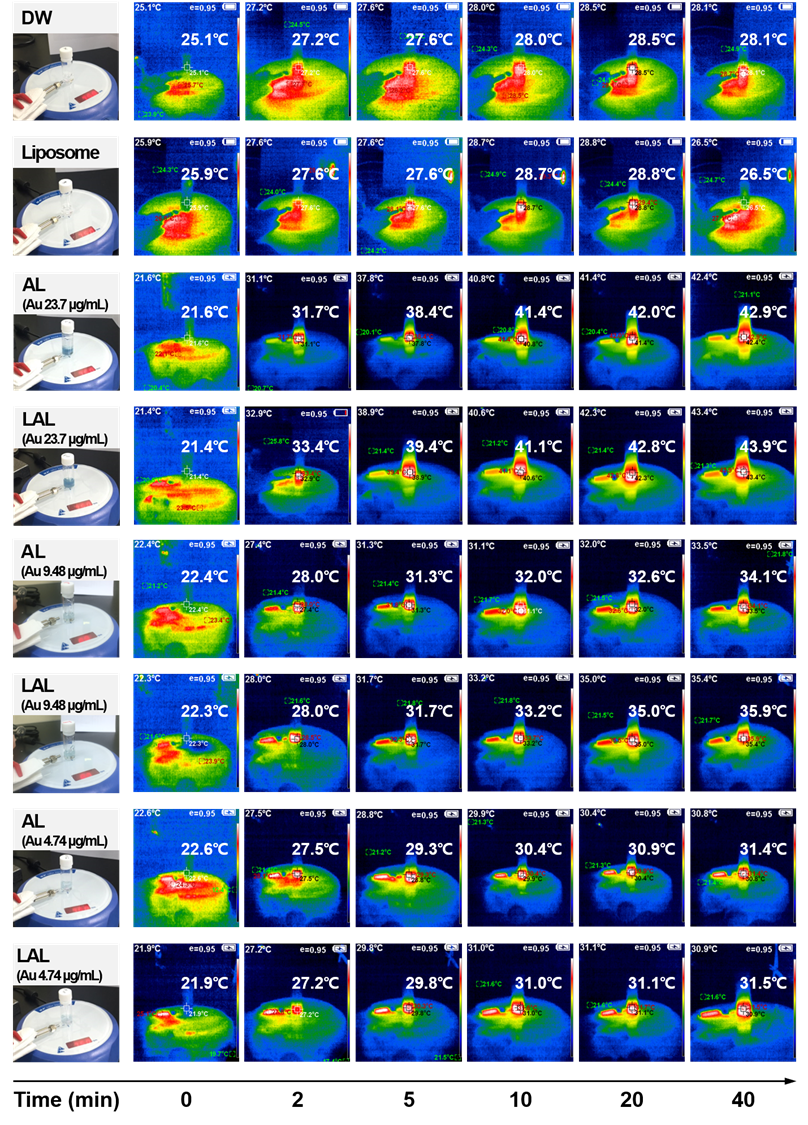
**

**Figure S4.** (a) Temperature evaluation of AL and LAL in RPMI 1640 media containing 10% FBS under 1 W 808-nm laser irradiation for 40 minutes (n = 3, mean ± s.d). The final Au concentrations of both AL and LAL were 23.7 μg mL^-1^. (b) Temperature changes of AL and LAL at 40 minutes laser irradiation (n = 3, mean ± s.d.). *: P < 0.05

**
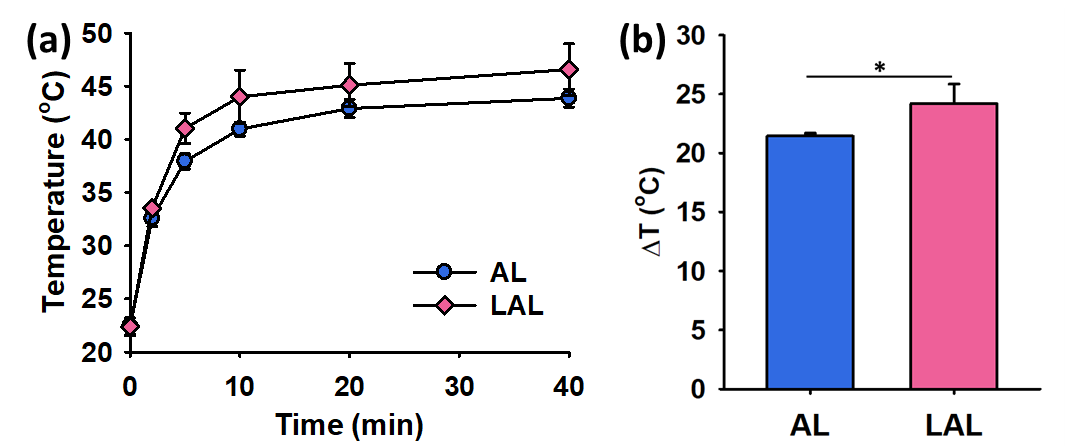
**

**Figure S5.** Ex vivo fluorescence images of tumors (Tu) and main organs (He: heart, Li: liver, SP: spleen, Ki: kidney, and Lu: lung) resected from each mouse. Upper row: DiR labeled LAL injected mice, lower row: DiR labeled AL injected mice.

**
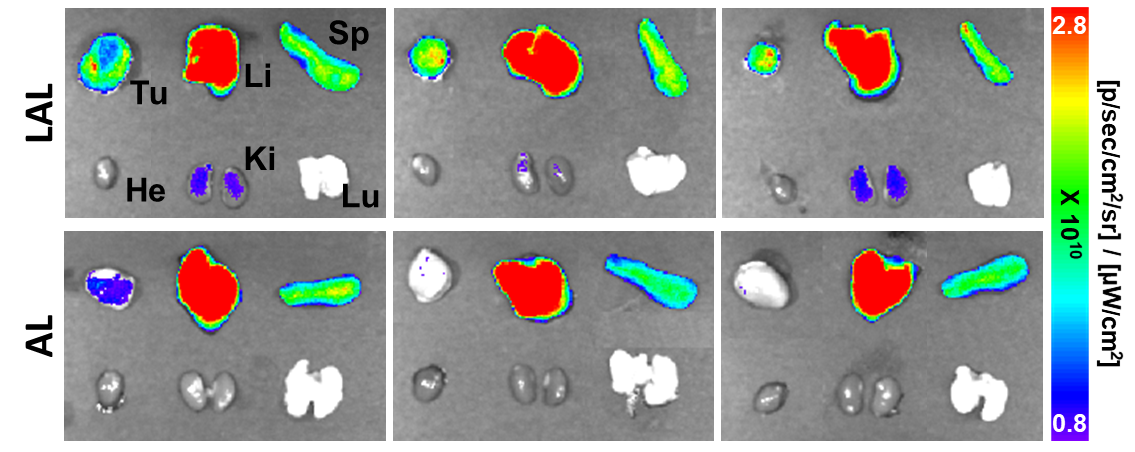
**

**Figure S6.** Photothermal effect on normal BALB/c-nude mice under the laser irradiation (808-nm, 2.5 W cm^-2^, 5 min) after subcutaneous injection of AL and LAL. (a) Thermal images of AL and LAL injected mice during laser irradiation. (b) Temperature comparison between AL and LAL at 5-minute (n = 3, mean ± s.d.). (c) Temperature changes of the site AL and LAL injected (n = 3, mean ± s.d.). * P < 0.05.

**
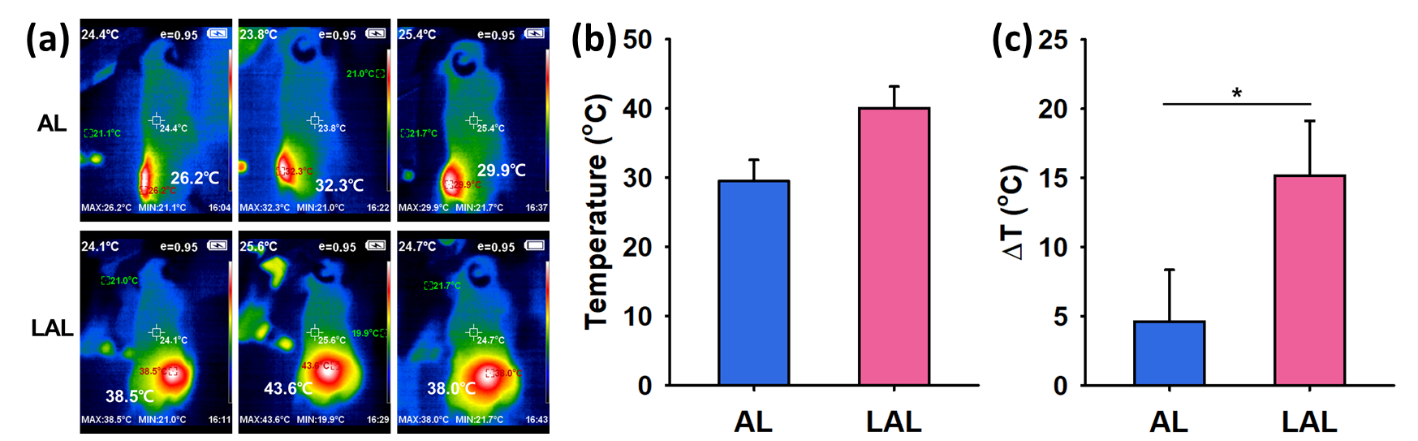
**

**Figure S7.** Thermal images of 4T1 tumor bearing BALB/c mice during the PTT with (a) NS, (b) AL, and (c) LAL treated groups.

**
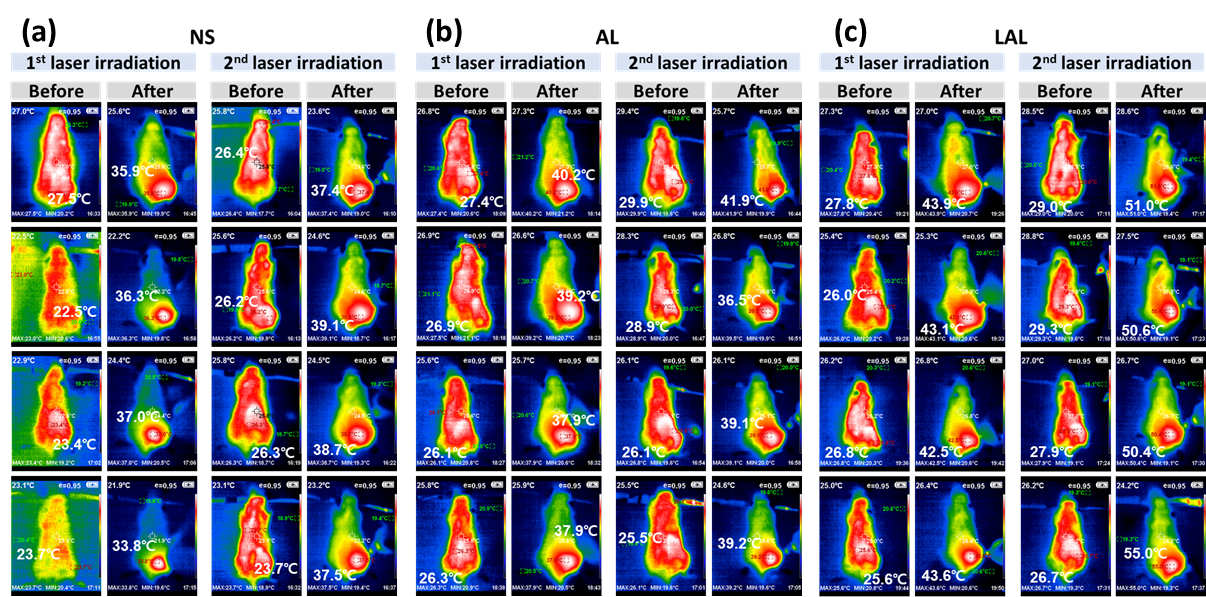
**

**Figure S8.** Representative tumor follow-up images (at day 1, 4, 11, and 18) of 4T1 tumor-bearing BALB/c mice after the PTT with NS, AL, and LAL treated groups.

**
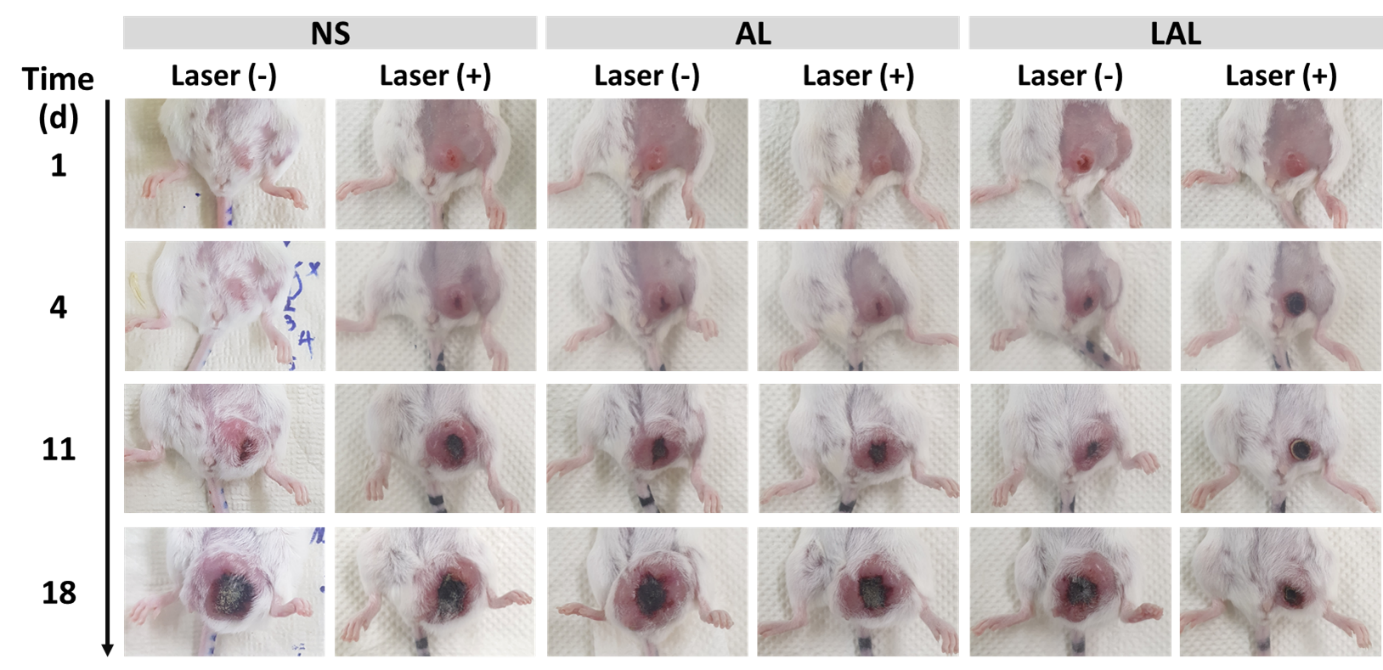
**

**Figure S9.** Histological analysis of H&E stained sectioned images of major organs (tumor, Tu; heart, He; liver, Li; spleen, Sp; kidneys, Ki; and muscle, Mu) after PTT experiment. All scale bars in the images are 100 μm.


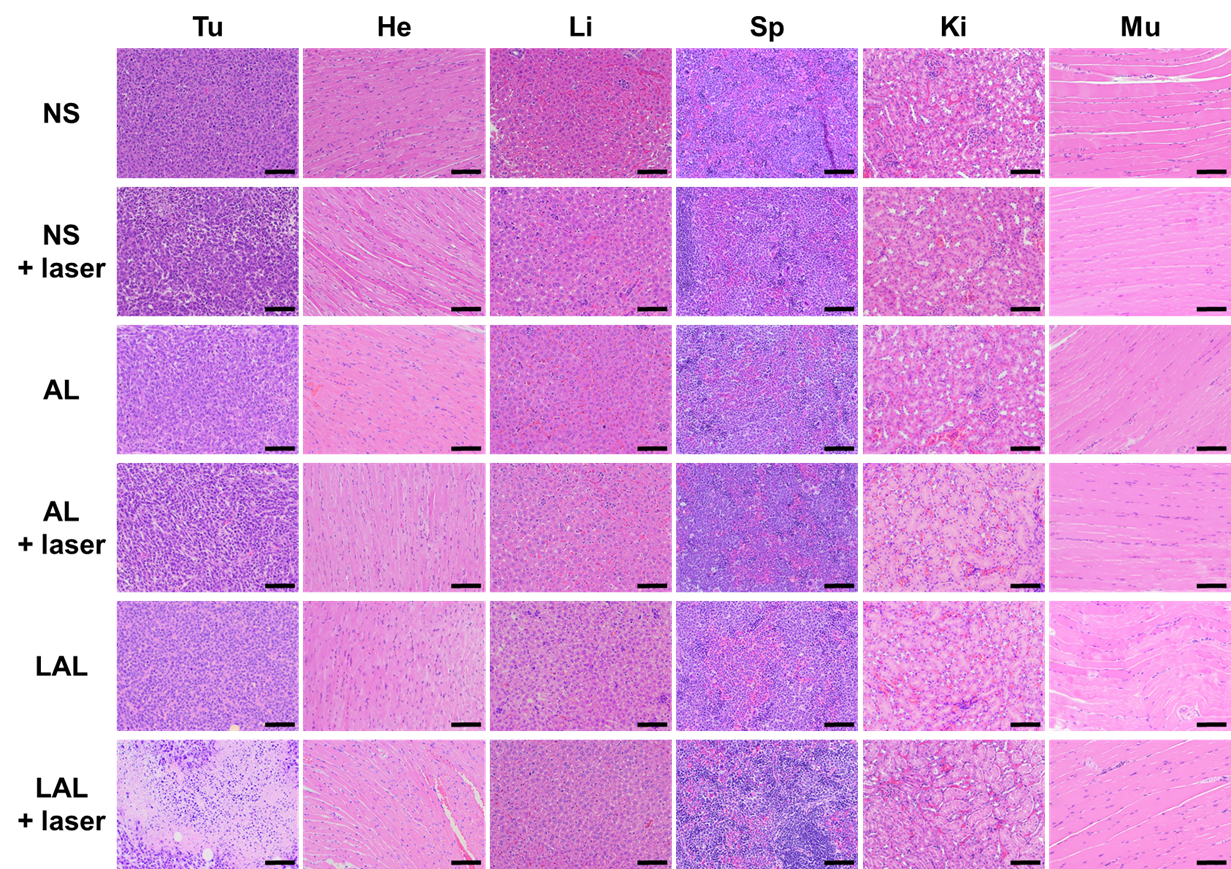


**Figure S10.** In vivo tumor target delivery efficacy profile of various NPs according to the review [1]. Red bar indicates LAL. The NPs delivery efficacy to tumors (%ID/g) were calculated the area under the curve of tumor uptake over time according to the review paper. The delivery efficacy of LAL by this calculation is 12.73 %ID/g and LAL is ranked 21^st^ among NPs from the highest.

**References**

1. Wilhelm S, Tavares AJ, Dai Q, Ohta S, Audet J, Dvorak HF, Chan WC. Analysis of nanoparticle delivery to tumours. Nat. rev. mater. 2016;1:1-12.
